# Plant Growth, Yield, and Quality of Bush Tea (*Athrixia phylicoides*) as Affected by Deficit Hidrico and Mulching

**DOI:** 10.3390/plants14121743

**Published:** 2025-06-06

**Authors:** Vhuhwavho Tshilidzi Ndou, Tafadzwanashe Mabhaudhi, Mangaliso Goge, Tshephiso Papo, Mzamo Shozi, Maanea Lonia Ramphinwa, Fhatuwani Nixwell Mudau

**Affiliations:** 1 School of Agricultural, Environmental Sciences and Earth Sciences, University of KwaZulu-Natal, Private Bag X01, Scottsville, Pietermaritzburg 3209, South Africa; 222128124@stu.ukzn.ac.za (V.T.N.); mabhaudhi@ukzn.ac.za (T.M.); 2 Centre on Climate Change and Planetary Health, London School of Hygiene and Tropical Medicine, London WC1E 7HT, UK; 3 School of Chemistry and Physics, University of KwaZulu-Natal, Private Bag X01, Scottsville, Pietermaritzburg 3209, South Africa; 217016445@stu.ukzn.ac.za (M.G.); papot@ukzn.ac.za (T.P.); 4 School of Chemistry and Physics, University of KwaZulu-Natal, Private Bag X54001, Durban 4000, South Africa; shozim2@ukzn.ac.za; 5 Department of Plant and Soil Science, Faculty of Sciences, Agriculture and Engineering, University of Venda, Private Bag X5050, Thohoyandou 0950, South Africa; maanea.ramphinwa@univen.ac.za

**Keywords:** physiological parameters, secondary metabolites, water regimes, sawdust mulch, black plastic mulch, ^1^H NMR, MS

## Abstract

Native to South Africa, Bush tea is a plant that thrives in various climates. Cultural practices such as mineral nutrition, fertigation, pruning, and harvesting have been shown to influence bush tea’s quality, growth, and yield. This study set out to determine the effects of mulching and deficit irrigation on the growth, yield, and quality of bush tea. Three deficit irrigation treatments (0%, 30%, and 100% Crop evapotranspiration (ETc) on field capacity) and three mulch treatments (sawdust, black plastic mulch, and no mulch) were included in a two-factor experiment, which was set up in a randomized complete block design (RCBD) with three replications. Physiological and growth parameters were taken every two weeks. The number of branches was counted, and measurements of chlorophyll content and the proportion of radiation intercepted by the canopy were recorded. Yield and secondary metabolites such as sugar residuals, fatty acids, and phenols of bush tea were determined after harvest. Growing bush tea under various water regimes showed that a 30% water regime significantly enhanced plant growth characteristics, including the proportion of intercepted radiation, plant height, and both fresh and dry weight. Furthermore, under different water regimes, sawdust improved plant growth in bush tea grown in the field. Black plastic mulch and a 0% water regime produced more compounds beneficial to health than tea treated with half or full irrigation. The extraction of data for Proton Nuclear Magnetic Resonance (NMR) and Mass Spectrometry analyses was conducted for quality components. Our study did not show any distinct structural differences in the tea under different water regimes or mulching. Flavones, phenols, diterpenes, and gardoside were some of the most abundant compounds found in bush tea using mass spectrometry. Principal Component Analysis was performed on the NMR spectral data across 27 samples of bush tea.

## 1. Introduction

Native to the provinces of KwaZulu-Natal, Mpumalanga, the Eastern Cape, and Limpopo, bush tea (*Athrixia phylicoides*) grows naturally in a variety of climates [1,2,3,4]. Traditional uses of tea leaves include medicinal uses [5], blood cleansing or purification, and the treatment of a variety of illnesses, including boils, acne, infected wounds, headaches, and voice loss [2,6].In addition, the Vhavenda people of north-eastern South Africa have used bush tea as an aphrodisiac [5], while the Zulu people of the Indian Ocean coast utilize a root infusion for treating coughs and as a purgative [5,7]. Moreover, bush tea is free of caffeine, unlike *Camellia sinensis* [6], and can serve as an alternative beverage [8]. Bush tea is currently harvested from the wild for its medicinal properties, indicating a potential to domesticate and commercialize the plant [1]. However, cultivating or domesticating bush tea for commercial purposes in South Africa faces challenges due to its reliance on wild harvesting, which may threaten the plant’s survival and lead to extinction. Therefore, it is crucial to adopt sustainable cultural practices for the domestication and commercialization of the plant, which require understanding how agricultural techniques, such as irrigation and mulching, impact plant growth, yield, and quality [9,10]. Previous studies demonstrated that the growth, yield, and chemical composition of bush tea are influenced by cultural practices, including mineral nutrition and fertigation [5,11], pruning [12,13], and harvesting [14], as well as environmental factors [3,8]. At present, information on how water regimes and mulching affect the growth, yield, and quality of bush tea is lacking.

Bush tea is a drought-tolerant plant compared to other tea cultivars [15] and has not yet been domesticated; it may be subjected to varying environmental conditions, particularly in terms of water availability. It has been reported that water availability affects photosynthesis, dry matter accumulation, and leaf and root growth [16,17]. Similar results were reported by [18,19], who discovered that water is crucial for supporting the vegetative and reproductive stages of plants, particularly in arid and semi-arid regions. Hence, variations in rainfall may significantly impact plant growth, yield, and quality in these regions [3]. These results also concur with those of Chiappero et al. [20], who emphasized the impact of water stress on various plant species, demonstrating its significance in relation to plant biomass, leaf area, and the production of secondary metabolites. In addition, medicinal plants grown under drought or water deficit conditions generally produce higher concentrations of active substances that protect them against free radicals and reactive oxygen species and prevent damage to the photosynthetic process [21]. Secondary metabolites, including phenolic compounds, have significant benefits for human health, in addition to plant protection [21]. Rumani et al. [22] investigated the effects of different water regimes on the growth, yield, and accumulation of secondary metabolites in bush tea grown under a protected tunnel environment. However, the influence of varying water regimes on the growth, yield, and nutrient quality of field-grown bush tea remains uncertain.

Mulching is a crucial agricultural practice used mainly for soil enhancement and environmental conservation [23]. It enhances the physical, biological, and chemical characteristics of the soil, helps regulate temperature fluctuations, retains more moisture in the soil, and enriches the soil with nutrients, all of which contribute to increased crop output and growth [24]. Prior research noted that mulching has been recommended as a management strategy in honeybush tea cultivation to retain soil moisture and control weeds [25]. Similarly, mulching in tea olive (*Osmanthus fragrans*) increased plant growth by increasing root activity and the soluble sugar and chlorophyll contents, as well as providing appropriate moisture and nutrients in the root zone [26]. The response of bush tea, in terms of growth, yield, and quality, to different types of mulching has not previously been documented. Therefore, the current study aims to determine the effects of mulching and deficit irrigation on the growth, yield, and quality of bush tea.

## 2. Materials and Methods

### 2.1. Experimental Site

**Figure 1:**
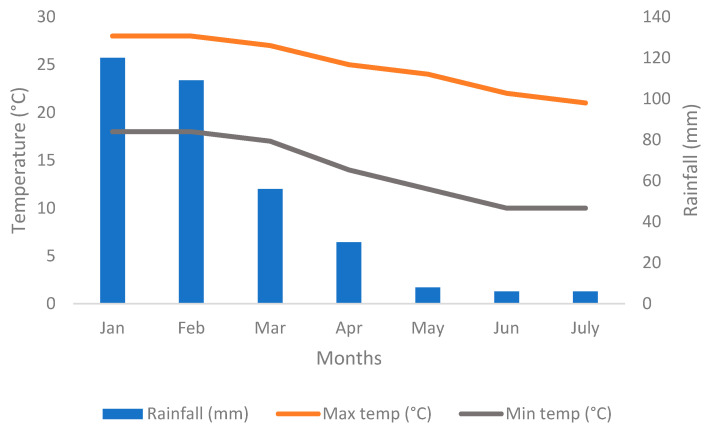
Average climatic data for the 2023 cropping season in Thohoyandou.

This study was conducted at the University of Venda (Univen), School of Agriculture experimental farm located approximately 22°35′14.0″ S and 30°15′50.3″ E. The climate is characterized by arid and semi-arid conditions, with an annual rainfall of ±500 mm per annum and temperatures that range from a minimum of 10 °C during winter to a maximum of 40 °C during summer. The site is characterized by deep, well-drained clay soil (Selolo et al. [27]). Weather data were supplied by the South African Weather Service (SAWS) for the 2023 cropping season. The weather data were collected from a weather station. Thohoyandou had monthly average maximum and minimum temperatures ranging from 25 to 28 °C and 10 to 18 °C, respectively, with average rainfall between 6 and 120 mm (Figure 1).

### 2.2. Plant Material Preparation

Cuttings from bush tea stems were gathered from the Univen farm. To encourage rapid root systems, 7–8 cm long plant cuttings were dipped in Starke Ayres DynarootTM No.2 hormone (0.3% IBA). Plants were planted using “true-to-name and type” material that was free of insect and disease damage. For 90 days beginning on 12 October 2022, stem cuttings were kept in round plastic pots in a lath house. A 2:1 mixture of sand and pine bark compost was used as the growth medium for propagation. Every day except on rainy days, the plants were irrigated. Cuttings that had roots were transplanted into 1 L bags and placed into a net shade house for a period of 60 days. Planting materials with 15 leaves or more were transplanted into the field on 20 March 2023.

### 2.3. Experimental Description

A two-factor experiment was arranged in a randomized complete block design (RCBD) with three replications. The field treatments had three water treatments/regimes of field capacity: Crop evapotranspiration under ideal conditions (ETc), i.e., 100% ETc, 30% ETc, and 0% ETc, and three mulch treatment, i.e., sawdust mulch (SM), black polyethylene plastic (BPM), and no mulch/control (NM). Also, a 100% water regime was used as a control treatment. The mulches covered the ridges on which the plants were planted and were spread evenly across the soil surface for uniform coverage. The plastic mulch was 6 mm thick. The experiment consisted of nine plots in total, with each plot measuring 4.2 m by 4.2 m, 1.5 m apart and 0.5 m intra-row spacing. Each plot consisted of four plant rows, each with 6 plants, making a total population of 24 plants per plot, and consequently, 216 plants/ha plant population.

Irrigation was carried out via sprinkler irrigation after transplanting bush tea cuttings to the field once every four days over a week. Plants were then subjected to drip irrigation 2 weeks after they had hardened. The deficit irrigation schedules per day were then 30% (1.8 h of irrigation) and 100% (6 h), conducted at developmental and late stages for the remaining plots, while mulching was applied one week after the plants were transplanted to the field. Irrigation was done 5 days per week when necessary. Nitrogen-phosphorus-potassium (NPK) fertilizer was applied in two equal splits (at transplanting and 4 weeks after transplanting) at a rate based on previous studies [28]. Metamidophos was applied at a rate of 10 mL per 20 L after each manual weeding to protect the plants against termites and aphids, as detailed in [4].

### 2.4. Data Collection

#### 2.4.1. Physiological Parameters

The proportion of intercepted radiation and the chlorophyll content were measured. The chlorophyll content was determined to be between 10H00 and 13H00 on each occasion from three plants in each plot using a chlorophyll content meter (CCM-200 plus; Opti-Sciences, Tyngsboro, MA, USA). These measurements were taken every two weeks.

The proportion of intercepted radiation was determined by measuring photosynthetically active radiation (PAR) above and below the canopy on various occasions, i.e., mostly at seven-day intervals in the winter. The measurements were taken between 11h00 and 13h00 on clear, cloudless days using an AccuPar LP-80 Ceptometer (model LP-80, Decagon Devices Ltd., Pullman, WA, USA), and the proportion of intercepted radiation was calculated as shown in Equation (1):

α = 1 − (P_A_/P_B_) (1)

where P_A_ = PAR above the canopy, P_B_ = PAR below the canopy, and α is the proportion of the intercepted radiation.

#### 2.4.2. Growth Parameters

Each plot had four rows, and data were collected for the winter season. Three plants in each experimental plot were harvested. A day before each harvest, the height of the plants was measured (from the soil surface to the tip of the topmost leaf, in cm) using a measuring tape. The number of branches was counted. Using a weighing balance, fresh weight and dry weight were recorded after each season’s harvest. The harvested plants were allowed to air dry for two months at ambient temperature. The proportion of dry matter content was obtained by expressing the dry weight percentage using Equation (2):

(2) $$Dry\;matter\;\%=\frac{Dry\;weight}{Fresh\;weight}\times 100$$

#### 2.4.3. Extraction for Proton Nuclear Magnetic Resonance and Mass Spectrometry Studies

A technique suggested in [29] was used to extract metabolites. In summary, the bush tea leaves were dried and crushed to a fine powder in a blender. Then, 2 g of the powder was extracted using 20 mL (1:1 *v*/*v*) of MeOH/DCM. The samples were rotated overnight on a shaker at 70 rpm. The mixture was then filtered into glass vials under a vacuum pump using 0.22 µm nylon membrane filters, and the solvent was evaporated. ^1^H NMR data were recorded on a Bruker Avance 400 MHz spectrometer at 303 K. The reported chemical shift values are in ppm relative to the solvent residual peaks for ^1^H NMR (C_3_D_6_O). Mass spectrometric data were obtained using an LC Premier micro-mass spectrometer model LCMS-2020.

#### 2.4.4. Microwave Digestion for Inductively Coupled Plasma-Optical Emission Spectroscopy (ICP-OEP) Analysis

First, 0.25 g of the powdered sample was weighed into microwave vessel tubes and dissolved in 10 mL nitric acid (70%) and 3 mL hydrogen peroxide. After digestion, the samples were transferred into 20 mL volumetric flasks and topped with ultra-pure water.

### 2.5. Data Analysis

The collected data were subjected to two-way analysis of variance (ANOVA) using GenStat Version 23.1 (VNS International, Maidstone, UK) to assess the treatment effects on physiological and growth parameters, i.e., plant height, number of leaves, number of branches, fresh and dry weight. The means were separated and compared using the Least Significant Difference Test (LSD) at a significance of *p* ≤ 0.05. Chemical composition was analyzed using a principal component analysis (PCA) and 3D visualization as a dimensionality-reduction method. The analyses were performed using Python (Version 3.13.3) with the Scikit-learn (Version 1.2.2) library for dimensionality reduction and Matplotlib (Version 3.7.1) along with Seaborn (Version 0.12.2) for data visualization. Data preparation was conducted using Pandas (Version 1.5.3) and Numpy (Version 1.24.2).

## 3. Results and Discussion

### 3.1. Influence of Water Regime and Mulching on Proportion of Intercepted Radiation

The plant growth parameters (number of branches, fresh weight, dry weight, and proportion of intercepted radiation) of field-grown bush tea under various water regimes varied significantly; see Table 1. Water excess negatively impacted plant performance, preventing growth and final production, just as water scarcity had significant effects on plant growth and development [30]. Accordingly, plants cultivated under a 30% water regime showed a larger percentage of intercepted radiation (67.95%), followed by plants grown under a 100% water regime (64.12%). The lowest IR was 60.18%, which was found in plants grown under a 0% water regime. The lowest intercepted radiation could be attributed to the reduced leaf area and the rolling up of wilted leaves due to water scarcity, resulting in the lowest plant growth in terms of branch count and fresh and dry weight. Similar findings in [31] suggested that water stress results in a decrease in photosynthetically active radiation (PAR) and radiation use efficiency (RUE) in maize crops. Black plastic mulch significantly had the highest intercepted radiation in this experiment, with 65.49%, a finding also supported by Abiodum et al. [32], who found that black polyethylene plastic resulted in high IR in tomatoes. This could have been because plastic mulches tend to increase soil temperatures [33], resulting in high growth and yield of crops.

### 3.2. Deficit Irrigation on Plant Growth and Yield of Bush Tea

Responses to different water regimes in terms of branch count, fresh weight, and dry weight followed a similar pattern to the effect of deficit irrigation on the proportion of IR: plants grown under the control (0%) water regime responded the least (17.00, 259.33 g/plant, and 167.50 g/plant), while those grown under 30% water regimes responded the most (45.44, 429 g/plant, and 282.73 g/plant) (see Table 1). According to Ashraf et al. [34], a lack of moisture causes plants’ turgor and water potential to drop, which has an adverse effect on their production and lowers their growth and yield. The results of the current study are consistent with a prior study [22], which found that superior bush tea production is achieved with 30% ETc compared to 100% and control. Nevertheless, as anticipated, 100% water treatment of field capacity produced the tallest plants, in agreement with prior research on the herb *Trachyspermum ammi* L [9]. Additionally, Mahlare et al. [35] found that honeybush tea plants that received regular irrigation consistently produced more than those in the other two groups (stressed and semi-stressed).

### 3.3. Influence of Mulching on Plant Growth and Yield of Bush Tea

The yields of bush tea plants planted with various mulching types showed no statistical significance relative to one another (Table 1). Nonetheless, mulching had an impact on the growth and yield of bush tea. The highest number of branches was found in plants that received sawdust mulching (33.67), followed by those in the control group (no mulching) with 30.22 branches, while the lowest number was observed in plants cultivated under black plastic. The findings of [32,36], who reported that sawdust enhanced the growth and yield of garlic and okra, respectively, are comparable to these results. Interestingly, plants exposed to no mulch produced the most fresh and dry mass (343.37 g/plant; 229.72 g/plant), followed by plants with black plastic mulch (343.22 g/plant; 229.34 g). This might have been because bush tea is a plant that grows naturally and can flourish in unfavourable conditions. Furthermore, bush tea has a high shoot water potential due to its drought tolerance, which enables it to maintain a moderate rate of photosynthesis and other metabolic processes while simultaneously maintaining high water usage efficiency [37].

### 3.4. Interactive Effect of Water Regimes and Mulching on Plant Growth and Yield of Bush Tea

We observed significant interaction between the effects of mulching and water regimes on the yield of bush tea. However, other parameters were not significant (Table 1). The highest yield was found with no mulch and a 30% water regime, while the lowest yield was found with sawdust mulch and a 0% water regime. Additionally, mulching showed no statistical significance on any of the growth parameters, while water regime showed statistical significance for number of branches. Notably, a 30% water regime favoured the field-grown bush tea’s morphological and physiological parameters. These results are in accordance with those of Rumani et al. [22], who concluded that a 30% water regime was the best water treatment for bush tea under a controlled environment. Black plastic mulch, however, resulted in the highest intercepted radiation, which may have been the reason for the highest fresh weight. Bush tea yield responses to the 30% water regime were directly proportional to IR and CCI. Moreover, when 30% IR was achieved with black plastic mulch, plant height and yield were enhanced. The observed vegetative growth improvement may be explained by the fact that that plastic mulches promote moisture conservation and availability, resulting in improved plant growth [38].

### 3.5. Influence of Water Regimes on Quality of Bush Tea

**Figure 2:**
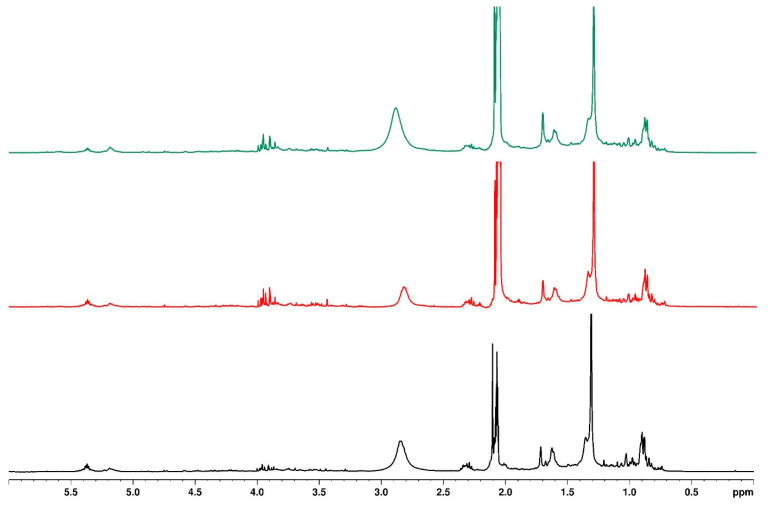
Overlaid ^1^H NMR spectrum for samples with no mulching and different water regimes (black—0%, red—30%, and green—100%), indicating the upfield region.

**Figure 3:**
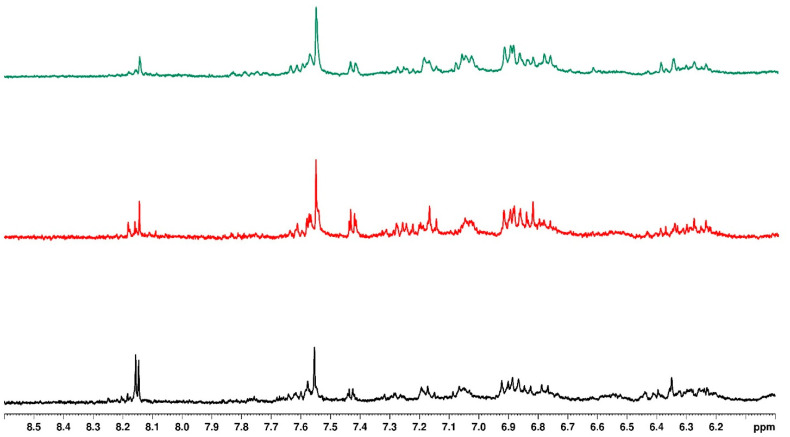
Overlaid ^1^H NMR spectrum for samples with no mulching and different water regimes (black—0%, red—30%, and green—100%), indicating the downfield region.

Figure 2 presents the upfield one-dimensional ^1^H NMR of bush tea samples under different water regimes with no mulching, while Figure 3 presents the downfield region. The upfield region indicates the fatty acids, sugar residues, and anomeric protons [39]. The downfield region indicates the aromatic protons and phenols. There were no distinct structural differences in the tea under different water regimes except for a doublet peak at 8.15 ppm for the tea with a 0% water regime. The same observation was made for the plastic mulch and sawdust-treated tea under different water regimes. While crop quality results from metabolic flux, which is attributed to compound biosynthesis and metabolism, crop yield is strongly correlated with physiological and growth characteristics [40]. Consequently, the capacity of bush tea to produce greater concentrations of secondary metabolites in situations of water deficit was likely the reason for the increased synthesis of biosynthetic chemicals seen under the 0% water regime [21].

### 3.6. Influence of Different Types of Mulching on Quality of Bush Tea

**Figure 4:**
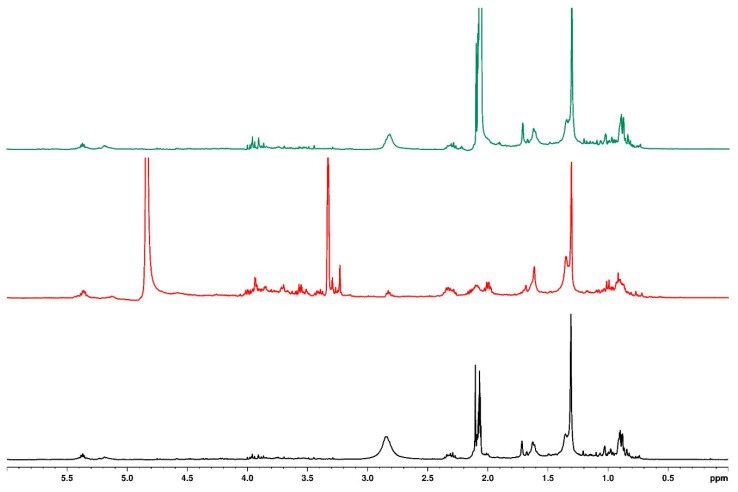
Overlaid ^1^H NMR spectrum for samples with different types of mulching and 0% water regime (black—no mulch, red—plastic mulch, and green—sawdust), indicating the upfield region.

Figure 4 represents the upfield one-dimensional ^1^H NMR of bush tea samples treated with different mulching types and the 0% water regime. The different peaks in the upfield region indicate a difference in structure for the tea treated with black plastic mulch compared to the no mulch and sawdust tea. The no-mulch and sawdust-treated teas had similar structures.

**Figure 5:**
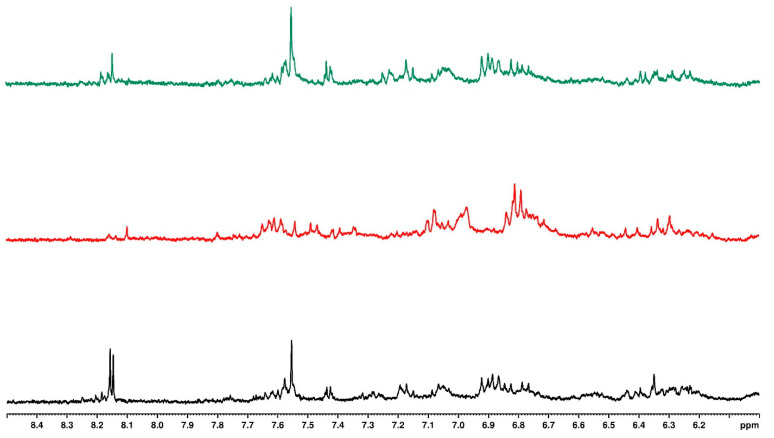
Overlaid ^1^H NMR spectrum for samples with different types of mulching and 100% water regime (black—no mulch, red—plastic mulch, and green—sawdust), indicating the downfield region.

Looking at the aromatic region in Figure 5, the structural compositions of the no-mulch and sawdust-treated teas were similar, except for the signal at 8.15 ppm, which appeared as a doublet in the no-mulch tea and a singlet in sawdust-treated tea. Similarly, the structural composition of black plastic mulching differed from the others.

Similar observations were made for the 30 and 100% water regimes. The structural features for no mulch and sawdust were identical, while the tea treated with black plastic mulch showed distinct differences in the NMR.

### 3.7. Mass Spectrometry

Table 2 shows the various metabolites found in the samples using chromatography in negative MS mode. The compounds were identified according to their retention times, masses, and molecular formulae under the various water treatment regimes. Communic acids were only found in samples with the 100% water treatment regime, which was consistent with what has been previously reported [22]. Communic acids belong to a naturally occurring group of compounds known as diterpenes; they have strong antimicrobial activity against pathogens [41]. Compounds that were common in all water regimes were 6-gingerol and flavones, namely, 5′-hydroxycudraflavone A and 5,4′,5′-trihydroxy-3,6,7,8,2′-pentamethoxyflavone. Both these compounds were also previously identified [22], with the latter compound’s presence being reported for the first time in that report. The compound 6-gingerol was present in several samples; this is a compound with versatile applications, as it is also found in ginger rhizomes, which are used as traditional medicines and in food [42]. Flavones, occurring mainly in green and black tea, have benefits for cardiovascular health [43]. Interestingly, for some samples, flavones were present together with gardoside, which is a type of glycoside. It has been reported that flavones in tea combine with sugars to form flavone glycosides [44]. Another important compound that was present in some samples was 4-caffeoylquinic acid. This compound belongs to a group of phenolic compounds found in tea, vegetables, fruits, herbs, wine, and coffee [45]. These compounds have been studied for their anti-inflammatory, anti-cancer, anti-bone disease, and anti-cardiovascular disease activities [46].

### 3.8. Principal Component Analysis of Bush Tea Samples

**Figure 6:**
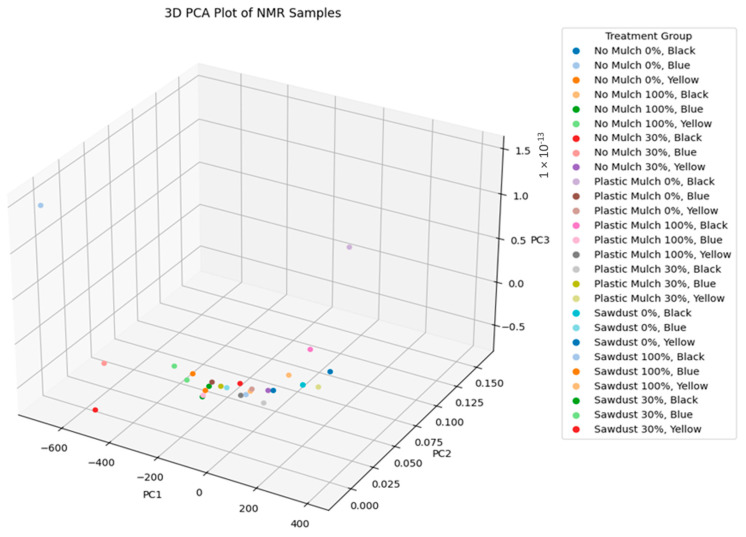
The 3D PCA plot displays clustering of the NMR spectral profiles of 27 samples subjected to different mulch treatments. The colors represent the number of plants sampled in all plots.

**Figure 7:**
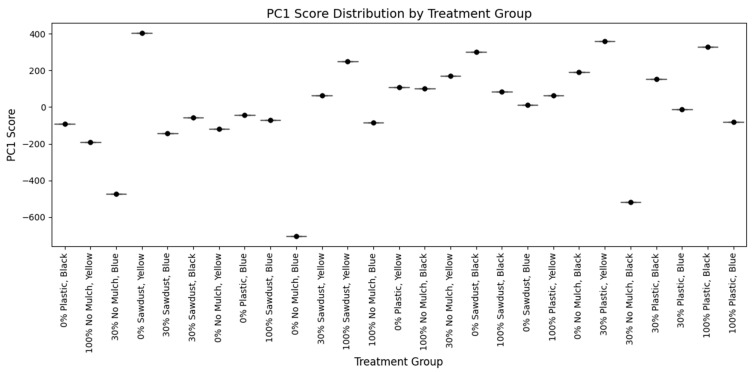
Principal Component 1 (PC1) score distribution plot showing the separation of NMR samples across 27 treatment groups combining mulching type and water regime treatments. The distribution along PC1 reflects the primary axis of metabolic variation, suggesting differential chemical responses to the applied treatments. The colors represent the number of plants sampled in all plots.

PCA (Figure 6 and Figure 7) was performed on the NMR spectral data from 27 samples. The first principal component (PC1) accounted for 99.9999985% of the total variation in the dataset, indicating a dominant source of variance among the samples. The second (PC2) and third (PC3) components explained only 1.49 × 10^−6^% and ~0% of the variation, respectively. The cumulative variance explained by the first three components was essentially 100%, with PC1 alone capturing nearly all meaningful variability.

The pronounced dominance of PC1 suggested a strong unidimensional pattern in the dataset, possibly due to a major underlying metabolite or concentration shift common across treatment groups. The second component and PC3 were likely capturing noise or minute experimental variability.

## 4. Conclusions

This study revealed that varying water regimes significantly affect the physiological parameters and secondary metabolite contents of field-grown bush tea. In our study, bush tea plants exposed to 30% ETa (crop water requirement) demonstrated enhanced growth and yield characteristics, e.g., the number of branches, fresh weight, dry weight, and proportion of intercepted radiation, compared to other treatments. The implementation of horticultural practices, such as the use of sawdust, led to an increase in both the fresh and dry weight of bush tea during production. Additionally, black plastic mulch demonstrated the ability to enhance plant quality by promoting the production of compounds that are beneficial to human health. *Athrixia phyllicoides* is a resilient crop that can thrive in harsh conditions, even without sufficient irrigation or horticultural practices. Therefore, combining 30% irrigation at field capacity with black plastic mulch may serve as the optimal approach to enhancing plant growth and for the production of secondary metabolites in bush tea.

## Figures and Tables

**Table 1:** Response of plant growth and yield under different types of mulching and water regimes and their interactions.

	Chlorophyll Content Index (CCI) (µmol m^−2^)	Dry Weight (g)	Fresh Weight (g)	Intercepted Radiation (%)	No. of Branches	Plant Height (cm)
Mulching						
Black Plastic	3.04 ^a^	229.34 ^a^	343.22 ^a^	65.49 ^a^	23.44 ^b^	65.49 ^a^
No Mulch	3.05 ^a^	229.72 ^a^	343.37 ^a^	64.02 ^a^	30.22 ^ab^	64.02 ^a^
Sawdust	2.82 ^a^	189.49 ^a^	342.13 ^a^	62.73 ^a^	33.67 ^a^	62.73 ^a^
Water Regime						
0%	2.80 ^a^	167.50 ^b^	259.33 ^b^	60.18 ^b^	17.00 ^b^	70.67 ^b^
30%	3.30 ^a^	283.73 ^a^	429.17 ^b^	67.95 ^a^	45.44 ^a^	71.67 ^b^
100%	2.81 ^a^	198.32 ^a^	340.22 ^b^	64.12 ^a^	25.89 ^a^	70.67 ^a^
*p*-value	ns	ns	ns	ns	*	ns
Mulching	ns	*	ns	ns	*	ns
Water Regime	*	*	**	***	**	ns
Mulching × Water Regime	ns	*	**	ns	**	ns
L.S. D	1.769	133.0	163.4	5.68	13.6	26.3
CV (%)	34.7	35.9	27.8	27.4	27.3	20.8

*, **, *** = significant at *p* < 0.05, *p* < 0.01, *p* < 0.0001 and ns = not significant (*p* > 0.05), CV = coefficient of variation, L.S.D = Least Significant Difference. Means within a column followed by the same letter indicate no significant difference. Means within a column followed by different letters indicate significant difference.

**Table 2:** Classification of various bioactive compounds from bush tea leaf extracts under varying water regimes.

Sample	Mass to Charge (*m*/*z*)	Retention Time (min)	Fragmentation Ion	Molecular Formula	Compound Name	Water Treatment		
						0%	30%	100%
A	353.1	7.75	354, 191, 179	C_16_H_18_O_9_	4-caffeoylquinic acid	✓		
	493.1	9.93	331, 210	C_23_H_25_O_12_^+^	malvidin-3-O-monoglucoside (oenin)			
AA	297.1	21.12	163	C_20_H_30_O_2_	communic acid			✓
	433.1	22.29	403, 225	C_25_H_22_O_7_	5′-hydroxycudraflavone A			
B	463.1	9.73	301	C_21_H_20_O_12_	quercetin-3′-O-glucoside			✓
	493.1	9.93	331, 210	C_23_H_25_O_12_^+^	malvidin-3-O-monoglucoside (oenin)			
C	353.1	7.50	135, 224	C_16_H_18_O_9_	4-caffeoylquinic acid		✓	
D	493.1	9.95	331	C_23_H_25_O_12_^+^	malvidin-3-O-monoglucoside (oenin)	✓		
E	293	29.88	276	C_17_H_26_O_4_	6-gingerol		✓	
F	293	29.88	276	C_17_H_26_O_4_	6-gingerol		✓	
G	353.1	7.50	354, 191, 179	C_16_H_18_O_9_	4-caffeoylquinic acid	✓		
	419.1	18.18	389, 214	C_20_H_20_O_10_	5,4′,5′-trihydroxy-3,6,7,8,2′-pentamethoxyflavone			
	433.1	22.29	403, 225	C_25_H_22_O_7_	5′-hydroxycudraflavone A			
H	433.1	22.29	403, 225	C_25_H_22_O_7_	5′-hydroxycudraflavone A	✓		
I	297	21.11	163, 117	C_16_H_22_O_10_	gardoside			✓
	293	29.88	276	C_17_H_26_O_4_	6-gingerol			
J	293	29.88	276	C_17_H_26_O_4_	6-gingerol	✓		
K	297	21.11	163, 117	C_16_H_22_O_10_	gardoside (glucoside)		✓	
	433.1	22.29	403, 225	C_25_H_22_O_7_	5′-hydroxycudraflavone A			
L	433.1	22.29	403, 225	C_25_H_22_O_7_	5′-hydroxycudraflavone A			✓
M	419.1	18.18	389, 212	C_20_H_20_O_10_	5,4′,5′-trihydroxy-3,6,7,8,2′-pentamethoxyflavone			✓
N	433.1	22.30	403, 214	C_25_H_22_O_7_	5′-hydroxycudraflavone A	✓		
O	293	29.88	276	C_17_H_26_O_4_	6-gingerol			✓
P	293	29.88	276	C_17_H_26_O_4_	6-gingerol		✓	
Q	353.1	7.76	191, 230	C_16_H_18_O_9_	4-caffeoylquinic acid	✓		
	419.1	18.18	389, 212	C_20_H_20_O_10_	5,4′,5′-trihydroxy-3,6,7,8,2′-pentamethoxyflavone			
R	419.1	18.18	389, 212	C_20_H_20_O_10_	5,4′,5′-trihydroxy-3,6,7,8,2′-pentamethoxyflavone			✓
	433.1	22.30	403, 225	C_25_H_22_O_7_	5′-hydroxycudraflavone A			
S	297	21.11	163, 117	C_16_H_22_O_10_	gardoside	✓		
	433.1	22.30	403, 225	C_25_H_22_O_7_	5′-hydroxycudraflavone A			
T	293	29.88	276	C_17_H_26_O_4_	6-gingerol			✓
U	419.1	18.18	389, 212	C_20_H_20_O_10_	5,4′,5′-trihydroxy-3,6,7,8,2′-pentamethoxyflavone	✓		
	433.1	22.30	403, 225	C_25_H_22_O_7_	5′-hydroxycudraflavone A			
V	293	29.88	276	C_17_H_26_O_4_	6-gingerol		✓	
W	293	29.88	276	C_17_H_26_O_4_	6-gingerol		✓	
X	293	29.88	276	C_17_H_26_O_4_	6-gingerol		✓	
Y	293	29.88	276	C_17_H_26_O_4_	6-gingerol		✓	
Z	Weak peaks on chromatogram							✓

## Data Availability

Data is contained within the article.
